# Late-onset Sheehan’s syndrome presenting with rhabdomyolysis and hyponatremia: a case report

**DOI:** 10.1186/1752-1947-7-227

**Published:** 2013-10-01

**Authors:** Maurizio Soresi, Giuseppe Brunori, Roberto Citarrella, Aurelia Banco, Antonino Zasa, Giovanna Di Bella, Lydia Giannitrapani

**Affiliations:** 1 Internal Medicine Unit, Biomedical Department of Internal Medicine and Subspecialties (DIBIMIS), University of Palermo, Palermo, Italy; 2 Section of Endocrinology, Biomedical Department of Internal Medicine and Subspecialties (DIBIMIS), University of Palermo, Palermo, Italy; 3 Department of Radiology, University of Palermo, Palermo, Italy

## Abstract

**Introduction:**

Hyponatremia associated with rhabdomyolysis is a rare event and a correct diagnostic approach is required to rule out this or other diseases as a primary cause and to avoid other complications resulting from a lack of appropriate treatment.

**Case presentation:**

A 64-year-old Caucasian woman presented to our facility with worsening fatigue, slurred speech, nausea and vomiting, and high serum levels of creatine kinase and myoglobin together with hyponatremia. Normal arterial blood gas analysis results, normal serum potassium levels, increased urine sodium levels, urine specific gravity of >1003N/m^3^ and low urine volume suggested an endocrine etiology. Her low cortisol and thyroid hormone serum levels suggested a pituitary disorder. A magnetic resonance imaging study showed atrophy of her pituitary gland. A more detailed study of our patient’s obstetric history revealed a post-partum hemorrhage 30 years earlier. She was diagnosed as having late-onset Sheehan’s syndrome and treated with hormone replacement therapy, which normalized her clinical picture.

**Conclusions:**

This case report shows that, in hyponatremia-associated rhabdomyolysis, an endocrinological origin should always be considered. This should include Sheehan’s syndrome as it can occur with late onset.

## Introduction

Hyponatremia is an event frequently found in clinical practice that physicians are often called upon to deal with, and it often results in complications including rhabdomyolysis [1]. In such cases in particular, a correct diagnostic approach is required to rule out this or other diseases as a primary cause and to avoid other complications resulting from a lack of appropriate treatment.

Here, we describe a case of rhabdomyolysis and hyponatremia with an infrequent endocrine etiology.

## Case presentation

A 64-year-old Caucasian woman was admitted to our Internal Medicine Unit for worsening fatigue, slurred speech, nausea and vomiting. In our Emergency Room she had been treated with saline solution infusion for hyponatremia (121mEq/L). Her clinical history showed nothing relevant until the age of 34, when she underwent a hysterectomy following post-partum complications; she had previously carried three pregnancies to term without complications. Thirty years later, a few months before her current admission, she began to experience fatigue, mental lethargy, myalgia and leg cramps, which were empirically treated with multivitamin supplements. She declined to undergo any further voluntary diagnostic evaluations, especially since the symptoms had partially improved. However, the onset of dysarthria, nausea and vomiting brought her to our Emergency Department.

As a cerebrovascular disease was suspected, a brain computed tomography (CT) scan without contrast medium was performed, which was within normal limits. The results of a physical examination revealed no neurological deficits except for the alteration in speech. Her skin was dry, her armpit hair was thinning and was reported by our patient to have been reduced for several years. Her hemodynamic and glycol-metabolic patterns were compensated. There were no clinical alterations in the thoracic or abdominal areas and no evidence of dehydration of the mucous membranes or peripheral edema. Table 1 shows the laboratory findings at presentation to hospital. Abdominal and thyroid ultrasound scans showed no alterations. The presence of myalgia and high creatine kinase (CK) and myoglobin values suggested rhabdomyolysis without renal involvement [2]. The lack of statin treatment or history of alcohol consumption together with the absence of trauma suggested that the rhabdomyolysis was related to hyponatremia or an immunological disorder. Specific autoantibody assay results, however, were negative. The association of hyponatremia with normal arterial blood gas analysis, normal serum potassium, normal urine sodium, urine specific gravity >1003N/m^3^ and urine osmolarity 174mOsm/L excluded polydipsia and suggested an endocrine etiology: hypocortisolism or hypothyroidism. Urine osmolality lower than serum values excluded syndrome of inappropriate anti-diuretic hormone hypersecretion (SIADH) [3,4]. Reduced levels of free circulating fractions of thyroid hormones associated with a thyroid-stimulating hormone (TSH) value inappropriately within the normal range indicated a possible thyroid and/or an anterior pituitary gland endocrine disorder [5,6]. As her cortisol levels were low, her adrenocorticotropic hormone (ACTH) level was measured, but this was within the normal range. In addition, low levels of prolactin (PRL), luteinizing hormone (LH) and follicle-stimulating hormone (FSH) left little doubt as to the diagnosis. A pituitary genesis also seemed plausible because of our patient’s medical history, which suggested late-onset Sheehan’s syndrome [7,8]. To confirm this hypothesis, our patient’s medical history leading to hysterectomy was further discussed with her. She could not recall the precise details but remembered bleeding that had required numerous blood transfusions. In addition, our patient was not able to give a detailed description of lactation and she had not breast fed on medical advice, probably due to the post-hysterectomy medical treatment.

**Table 1:** Laboratory test results at presentation to hospital and at one and two weeks later

	**Presentation**	**Week one**	**Week two**	**Reference values**
Blood urea nitrogen, mg/dL	28	20	33	10 to 50
Creatinine, mg/dL	0.83	0.78	0.9	0.6 to 1.0
Sodium, mEq/L	129	132	138	135 to 145
Potassium, mEq/L	4.75	4.6	4.5	3.5 to 5
Chlorine, mEq/L	92	98	105	95 to 108
Glucose, mg/dL	61	70	75	70 to 110
Cholesterol, mg/dL	310	300	222	<180
High-density lipoprotein, mg/dL	45	43	49	40 to 85
Triglycerides, mg/dL	172	180	150	<190
Glutamic oxaloacetic transaminase, U/L	123	58	41	7 to 34
Glutamic pyruvic transaminase, U/L	60	29	28	8 to 50
Creatine kinase, U/L	1377	678	292	40 to 300
Lactate dehydrogenase, U/L	1023	950	605	240 to 450
Myoglobin, ng/mL	1150	380	88	<90
Uric acid, mg/dL	2.9	2.4	2.7	2.3 to 6
Plasma osmolarity, mOsm/L	277	280	292	280 to 300
Urine:				
Specific gravity, N/m^3^	1005	1009	1010	1003 to 1030
Osmolarity, mOsm/L	175	315	350	105 to 1050
pH	7	7.5	6.5	4.5 to 7
Urine sodium, mEq/L/24 hours	93	142	130	50 to 250
Urine potassium, mEq/L/24 hours	16	30	45	30 to 110
Hormones:				
Free tri-iodothyronine, pg/mL	0.6		1	2.0 to 4.4
Free thyroxine, ng/dL	0.06		0.21	0.93 to 1.7
Thyroid-stimulating hormone, uIU/mL	1.56		2.19	0.27 to 4.2
Adrenocorticotropic hormone, pg/mL (8.00 am)	17		-	7.2 to 63.3
Cortisol, μg/mL	1.4		-	6.2 to 19
Prolactin, ng/mL	1.41		-	4.79 to 23.3
Follicle-stimulating hormone, mIU/mL	7.04		-	25.8 to 134
Luteinizing hormone, mIU/mL	3.0		-	7.7 to 58.5

One-week values are without hormone therapy. Two-week values are with cortisone acetate therapy.

**Figure 1:**
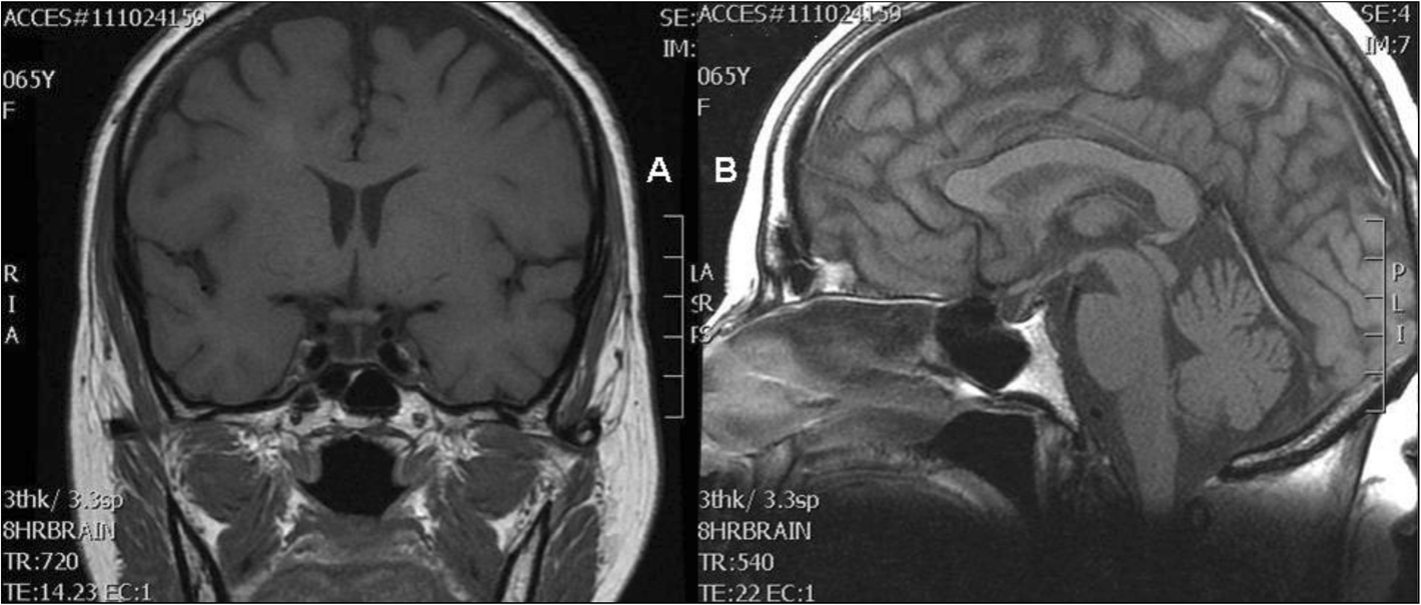
**Coronal (A) and sagittal (B) T1-weighted magnetic resonance imaging.** Images show marked diminution in the size of the pituitary gland. The pituitary fossa appears normal in size. The pituitary stalk is well seen and correctly oriented.

Magnetic resonance imaging with contrast medium showed a marked decrease in pituitary gland size (Figure 1) with signs of a chronic deficit in frontal cortical flow and a small cortical malacia. Cortisone acetate therapy was started at a dose of 25mg in the morning and 12.5mg after six hours. After the first two weeks l-thyroxin replacement treatment at a dose of 1.6μg/kg body weight was added. Her clinical symptoms and laboratory abnormalities resolved and serum sodium levels and myolysis indices normalized after five days. Given the age of our patient, estrogen replacement therapy was not necessary.

## Discussion

Atrophy of the pituitary gland was interpreted in our patient’s case as a likely outcome of late-onset Sheehan’s syndrome, a consequence of a post-partum hemorrhage that our patient was able to recall.

Sheehan first described pituitary infarction and panhypopituitarism after a post-partum hemorrhage in 1938, but the mechanism of ischemia is still not entirely clear. Hypotension and subsequent pituitary arterial vasospasm seem to compromise blood perfusion in the pituitary gland, causing its necrosis. Signs of hypopituitarism are reported in 32 percent of women with severe post-partum hemorrhage. The rapidity of onset and degree of pituitary insufficiency depend on the extent of the damage. The gland, however, has a high reserve and more than 75 percent of the pituitary needs to be damaged before clinical manifestations are evident. The first of these appears to be the absence of lactation after delivery. Although a small percentage of patients with Sheehan’s syndrome present with severe hypopituitarism immediately after labor, in many patients it remains undiagnosed and the pituitary failure is only recognized and treated after many years [7,8], as in our patient’s case. CT and magnetic resonance imaging (MRI) in most cases indicate an etiologic diagnosis. Imaging results show an empty sella and an atrophic gland, as in our patient. The MRI results also showed signs of chronic flow deficits, which could to some extent justify the syndrome manifestation as a result of the progressive loss of residual functional reserve. Another condition considered was lymphocytic hypophysitis, related to pregnancy in most of the women studied. There are in fact many similarities between Sheehan’s syndrome and lymphocytic hypophysitis, which is an autoimmune disease seen in both sexes. However, in our patient’s case the absence of clinical signs and serological markers of autoimmunity, together with the obstetric history and the imaging results, excluded this diagnosis [9].

A diagnosis of secondary hypothyroidism was suggested by the low free tri-iodothyronine (fT3) and free thyroxine (fT4) levels, with values inappropriately within the normal range or below the standard in the case of TSH [6]; but fT3 and fT4 levels were almost 0, excluding euthyroid sick syndrome [10].

Although secondary hypothyroidism appears to be a rare condition it must always be considered when there is a similar hormonal pattern, particularly in younger individuals without acute or chronic thyroid disease. Our patient’s case demonstrates the importance of a correct differential diagnosis that does not overlook medical history clues or signs that seem to be of minor importance. However, it also emphasizes the essential role of state-of-the-art laboratory tests and diagnostic imaging. Specifically, comparative assessments of blood and urine tests allowed us to correctly choose between the many causes of hyponatremia. The hormonal evaluation suggested a solely pituitary genesis and the accuracy of MRI indicated the organic etiology of the syndrome event. Regarding the therapeutic approach, replacement therapy re-established a satisfactory physical and mental well-being, although recent cohort studies have shown an increased mortality in patients with hypopituitarism despite treatment [11]. It must be underlined that before the hormone replacement therapy was started there was a partial resolution of rhabdomyolysis, as shown by a modest decrease in the necrosis indices, likely due to the correction of hyponatremia following the administration of hypersaline solutions. Hyponatremia is one of the spectrum of conditions that can lead to rhabdomyolysis, and in our patient’s case there may have also been an electrolyte imbalance in addition to the endocrine genesis. The literature offers various data to support this hypothesis [1]. However, only with the hormone replacement therapy was it possible to obtain a fluid and electrolyte balance and a reduction in the indices of cytolysis and rhabdomyolysis to within normal values.

Several mechanisms are responsible for hyponatremia in Sheehan’s syndrome: hypothyroidism, glucocorticoid deficiency, volume depletion, and SIADH. Moreover, both adrenal insufficiency and hypothyroidism can lead to myopathy.

In our patient, steroid therapy alone normalized her serum sodium, CK and myoglobin levels. Therefore, we believe that adrenal insufficiency was the main cause of the hyponatremia and rhabdomyolysis. However, an association between hypothyroidism and severe hyponatremia is also known, mainly due to an increased release of anti-diuretic hormone (ADH) caused by a reduced sensitivity of the osmoreceptors to reductions in plasma osmolality [12].

With regard to metabolic alterations, in particular hyperlipemia, treatment with thyroid hormones completely corrected hypertriglyceridemia and reduced the hypercholesterolemia recorded at entrance.

Our patient has now been placed on a six-month out-patient follow-up to monitor her clinical status and to allow any necessary adjustments to the treatment.

## Conclusions

The present report shows that in hyponatremia-associated rhabdomyolysis an endocrinological origin has always to be considered, including Sheehan’s syndrome as it can occur with late onset.

Several diseases can be associated with hyponatremia and rhabdomyolysis with a poor prognosis. The present work suggests the need for an adequate diagnostic approach based on simple and inexpensive tests (including serum and urine laboratory tests along with arterial blood gas analysis) that focus on the early identification of the cause and its correct treatment.

## Consent

Written informed consent was obtained from the patient for publication of this case report and any accompanying images. A copy of the written consent is available for review by the Editor-in-Chief of this journal.

## Abbreviations

ACTH: Adrenocorticotropic hormone; ADH: Anti-diuretic hormone; CK: Creatine phosphokinase; CT: Computed tomography; FSH: Follicle-stimulating hormone; fT3: Free triiodothyronine; fT4: Free thyroxine; LH: Luteinizing hormone; PRL: Prolactin; SIADH: Syndrome of inappropriate anti-diuretic hormone hypersecretion; TSH: Thyroid-stimulating hormone.

## Competing interests

The authors declare that they have no competing interests.

## Authors’ contributions

SM performed the diagnosis and was a major contributor in writing the manuscript. BG followed our patient during her recovery. CR evaluated and followed our patient from an endocrinological point of view. BA performed the magnetic resonance imaging study. ZA followed our patient during the follow-up period. DBG followed our patient as her general practitioner. GL analyzed and interpreted the data from our patient, and revised the literature by contributing to writing the manuscript. All authors read and approved the final manuscript.
